# Beat AML genetic risk stratification model in a cohort of older VEN/HMA-treated patients with AML

**DOI:** 10.1016/j.bneo.2025.100125

**Published:** 2025-06-03

**Authors:** Fieke W. Hoff, Ashley O. Yocum, Uma M. Borate, Alice S. Mims, John C. Byrd, Yazan F. Madanat

**Affiliations:** 1 National Heart, Lung, and Blood Institute, National Institutes of Health, Bethesda, MD; 2 Leukemia & Lymphoma Society, Rye Brook, NY; 3 Division of Hematology, Department of Medicine, The Ohio State University, Columbus, OH; 4 Department of Internal Medicine, University of Cincinnati, Cincinnati, OH; 5 Department of Internal Medicine, The University of Texas Southwestern Medical Center, Dallas, TX

**TO THE EDITOR:**

Acute myeloid leukemia (AML) is a disease of the older patient population, with a median age of 69 years.^1^^,^^2^ The standard of care for patients who are not eligible for intensive chemotherapy due to age or comorbidities generally consists of a hypomethylating agent (HMA) combined with the B-cell lymphoma 2 (BCL2)-inhibitor venetoclax (VEN).^3^^,^^4^ However, the currently used European LeukemiaNet (ELN) 2022 classification for risk stratifying newly diagnosed patients with AML was developed largely based on younger patients and/or those treated with intensive chemotherapy, with or without allogeneic stem cell transplantation. Several retrospective analyses have shown that ELN 2022 performs suboptimally in patients treated with HMA + VEN or other lower-intensity therapies.^5^, ^6^, ^7^, ^8^

To better stratify patients treated with HMA + VEN, a new 4-gene molecular prognostic risk signature (mPRS) has been proposed, incorporating gene mutations in *FLT3*-ITD, *KRAS*, *NRAS* (all intermediate risk or intermediate benefit), and *TP53* (adverse risk or lower benefit). This classification was established based on a pooled cohort of 279 patients enrolled in the phase 3 VIALE-A study (NCT02993523) and phase 1b study (NCT02203773) treated with HMA + VEN. The model showed a superior ability to predict overall survival (OS) compared to ELN 2022 in 3 independent cohorts: 1 single center including 159 patients,^5^ a multicenter analysis including 279 patients,^8^ and a multicenter cohort including 430 patients,^7^ all treated with HMA + VEN. The ELN also proposed a genetic risk classification framework (“ELN 2024 less intensive”) for patients receiving less-intensive HMA-based therapy enrolled in the VIALE-A and the AGILE phase 3 (NCT03173248) studies.^9^ Based on the observation that, in patients with *NPM1*- or *IDH2*-mutated AML, a negative prognosis was observed in the setting of concomitant signaling gene mutations, *NPM1* and *IDH2* were considered favorable risk in the absence of *NRAS*, *KRAS*, and *FLT3*-ITD. A *DDX41* mutation was considered favorable risk, as was *IDH1* in patients treated with azacitidine + ivosidenib.

However, because pertinent questions remained regarding the impact of other gene mutations affecting survival, a refined ELN 2024 risk stratification was proposed, which considered *KRAS*, *PTPN11*, and *TP53* mutations as adverse risk and *NPM1*, *IDH*, and *DDX41* mutations as favorable risk, showing an even better ability to predict risk.^7^ Importantly, mutations not otherwise classified were considered intermediate risk, whereas they were classified as favorable risk in ELN 2024.

Given the importance and challenges of accurate risk stratification, we conducted a retrospective analysis using a multicenter cohort of 238 patients with newly diagnosed AML aged ≥60 years enrolled in the Beat AML clinical trial (NCT03013998) and treated with HMA + VEN. Our analysis aimed to validate both the 4-gene mPRS and the refined ELN 2024 risk stratification models in an independent cohort, because these 2 models were established exclusively based on patients treated with HMA + VEN, whereas ELN 2024 also included patients treated with azacitidine + ivosidenib. Our cohort had a median age of 73 years (range, 60-89), 39.9% were female, and the median OS was 14.1 months (95% confidence interval, 12.10-18.12), similar to that observed in VIALE-A (supplemental Figure 1). The baseline cytogenetics and frequently mutated genes are listed in supplemental Table 1. Patients had favorable-, intermediate-, and adverse-risk gene mutations in 54%, 25%, and 21%, respectively, per 4-gene mPRS, and in 18%, 46%, and 37%, respectively, per the refined ELN 2024 (supplemental Table 2). Although data on *DDX41*, a favorable-risk gene mutation in the refined ELN 2024 classification, were not available in our cohort, we believe that this limitation likely has minimal impact on the overall risk stratification; only 8 patients harbored *DDX41* mutation in one of the published cohorts, and no data were reported on its prevalence in any of the other patient cohorts. Furthermore, *DDX41* did not have a significant prognostic impact in the univariate analysis by Lachowiez et al.^7^

**Figure 1.**
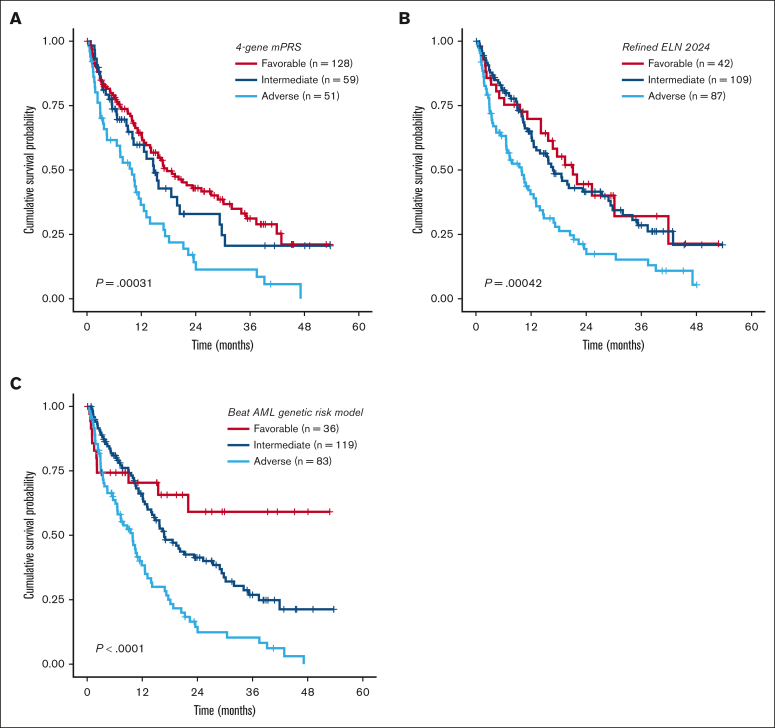
**Kaplan-Meier OS analysis for patients with newly diagnosed AML aged ≥60 years treated with HMA + VEN.** (A-C) OS analysis stratified by the 4-gene mPRS, refined ELN 2024, and the Beat AML genomic risk model.

**Figure 2.**
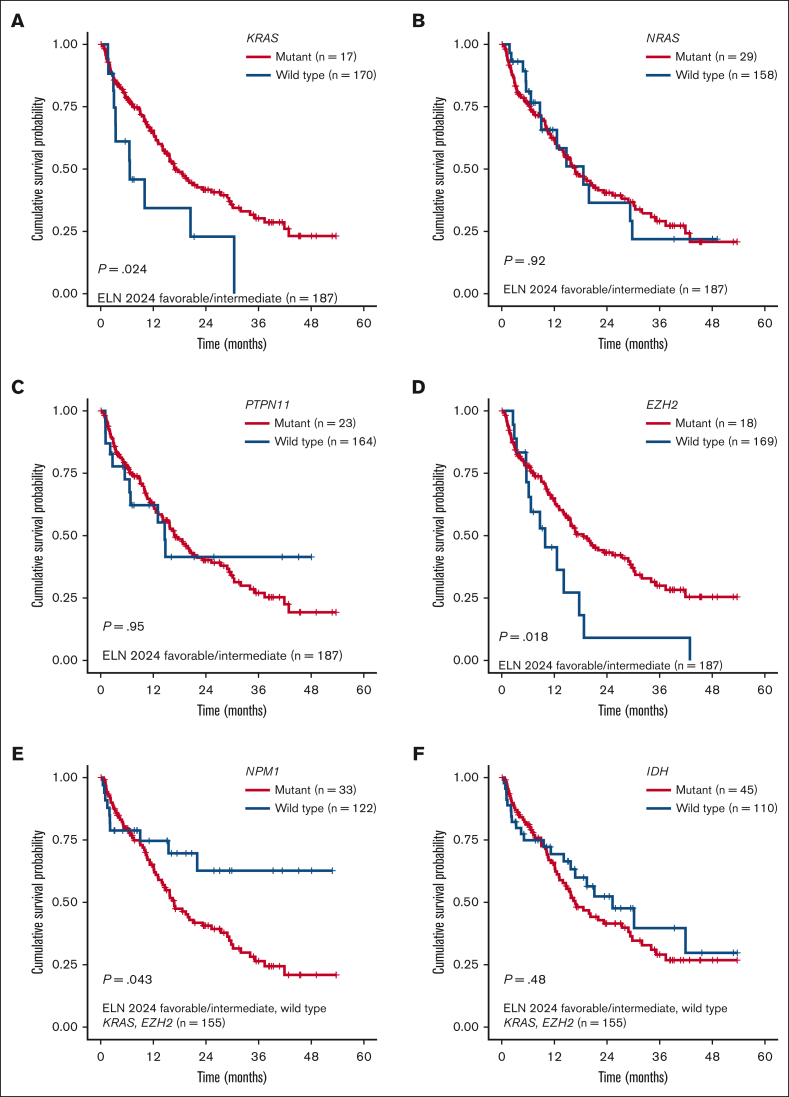
**Kaplan-Meier OS analysis for patients with newly diagnosed AML aged ≥60 years.** (A-D) Survival analysis stratified by *KRAS*, *NRAS*, *PTPN11*, and *EZH2* mutations among patients with ELN 2024 favorable- or intermediate-risk AML. (E-F) Survival analysis stratified by *NPM1* and *IDH* mutations among patients with ELN 2024 favorable- or intermediate-risk AML with *KRAS* and *EZH2* wild type.

Overall, both models were prognostic for OS in our cohort, with a marginally higher OS discrimination ability for the refined ELN 2024 than the mPRS (concordance index [C-index], 0.573 for the mPRS vs 0.588 for the revised ELN 2024; Figure 1A-B). Because the refined ELN 2024 classified *KRAS* and *PTPN11* as adverse-risk genes, we then sought to evaluate the prognostic impact of RAS pathway genes (*NRAS*, *KRAS*, and *PTPN11*) in our cohort. Within the mPRS favorable- and intermediate-risk groups (ie, all patients excluding *TP53* mutant), *KRAS*-mutated patients had inferior OS (*P* = .024; median OS, 18.6 vs 9.9 months), whereas *NRAS* (*P* = .92) and *PTPN11* (*P* = .95) were not prognostic (Figure 2A-C). In addition, 3 other genes were prognostic using univariate Cox regression analysis, all involved in epigenetic regulation: *EZH2, TET2*, and *DNMT3A* (supplemental Table 3).

*EZH2* mutations were present in 9.6% of patients in the favorable- and intermediate-risk mPRS groups and corresponded with shorter OS than *EZH2* wild type (median OS, 6.1 vs 14.8 months; *P* = .018; Figure 2D). *EZH2* encodes for the histone methyltransferase and is a critical component of the Polycomb repressive complex 2. It is also one of the pathologic myelodysplasia-related gene mutations that is considered adverse risk in 2022 ELN, and the presence of *EZH2* confers a diagnosis of myelodysplastic syndrome/AML rather than myelodysplastic syndrome for patients with ≥10% blasts per Internation Consensus Classification.^10^^,^^11^ Because the impact of *TET2* and *DNMT3A* mutations remains controversial in the literature and they commonly have co-occurring mutations driving leukemogenesis, we did not further explore the impact of these mutations in this cohort.

Next, we evaluated the impact of *NPM1* and *IDH* mutations, excluding patients with *TP53*, *KRAS*, and/or *EZH2* mutations. *NPM1* mutation was present in 21% of patients and was associated with a favorable prognosis compared to *NPM1* wild type (median OS, 21.99 vs 13.9 months; *P* = .043; Figure 2E). *IDH* showed a nonsignificant higher median OS (*P* = .48; Figure 2F). Risk stratification groups were then reclassified as favorable risk (ie, mutant *NPM1* and *DDX41* and wild-type *TP53*, *KRAS*, and *EZH2*), intermediate risk (not classified as favorable or adverse risk), and adverse risk (mutant *TP53*, *KRAS*, and *EZH2*) as the “Beat AML genetic risk model” for HMA + VEN (supplemental Figure 2), with a median OS of not reached, 16.8 months, and 9.9 months for favorable, intermediate, and adverse risk, respectively (*P* < .001; C-index, 0.586; Figure 1C). The median OS for favorable risk was not reached, compared to 17.7 and 21.1 months for the mPRS and the refined ELN 2024 models, respectively.

In conclusion, the current ELN 2022 does not apply to patients treated with HMA + VEN therapy, and new models have been proposed to better predict risk in this population. Although our study validated the utility of previously proposed models, we demonstrated that they do not yet predict outcomes for all patients. Larger prospective studies are needed that build upon these models to further refine risk classification in patients treated with HMA + VEN.

Informed consent was obtained in accordance with the Declaration of Helsinki.

**Conflict-of-interest disclosure:** U.M.B. has been a consultant for 10.13039/100004328Genentech, 10.13039/501100022274Daiichi Sankyo, 10.13039/100007723Takeda, 10.13039/100004319Pfizer, 10.13039/100006483AbbVie/10.13039/100004328Genentech, and 10.13039/100004336Novartis. A.S.M. has served on the advisory boards of 10.13039/100006483AbbVie/10.13039/100004328Genentech, 10.13039/100004336Novartis, Ryvu Therapeutics, Rigel Pharmaceuticals, Treadwell Therapeutics, and 10.13039/100016346Foghorn Therapeutics. J.C.B. is a current equity holder in Vincerx Pharma Inc (a publicly traded company), Eilean Therapeutics, and Kurome Therapeutics; holds membership on board of directors or advisory committees for Vincerx, Newave, Eilean Therapeutics, Kartos, and 10.13039/501100003951Orange Grove Bio; and received consultancy fees and honoraria from 10.13039/100004336Novartis, Trillium, 10.13039/100004324Astellas, 10.13039/100004325AstraZeneca, 10.13039/100014491Pharmacyclics, and 10.13039/100018201Syndax. Y.F.M. has received honoraria/consulting fees from 10.13039/100002491Bristol Myers Squibb, Kura Oncology, Blueprint Medicines, Geron, OncLive, MD Education, The Video Journal of Hematology and Hematological Oncology, and MedscapeLIVE!; advisory board fees and honoraria from 10.13039/501100019266Sierra Oncology, Stemline Therapeutics, Blueprint Medicines, MorphoSys, Taiho Oncology, SOBI, Rigel Pharmaceuticals, Geron, 10.13039/100016952Cogent Biosciences, and 10.13039/100004336Novartis; and travel reimbursement from Blueprint Medicines, MD Education, and MorphoSys. The remaining authors declare no competing financial interests.
