# Hoff FW, Yocum AO, Borate UM, et al. Beat AML genetic risk stratification model in a cohort of older VEN/HMA-treated patients with AML. *Blood Neoplasia.* 2025;2(3):100125.

**DOI:** 10.1016/j.bneo.2025.100169

**Published:** 2025-11-09

On page 3, the colors in the legends for Figure 2A-F are transposed. Blue should correspond to the mutated cases, and red should represent wild type. The corrected Figure 2A-F is shown below.

**Figure 2.**
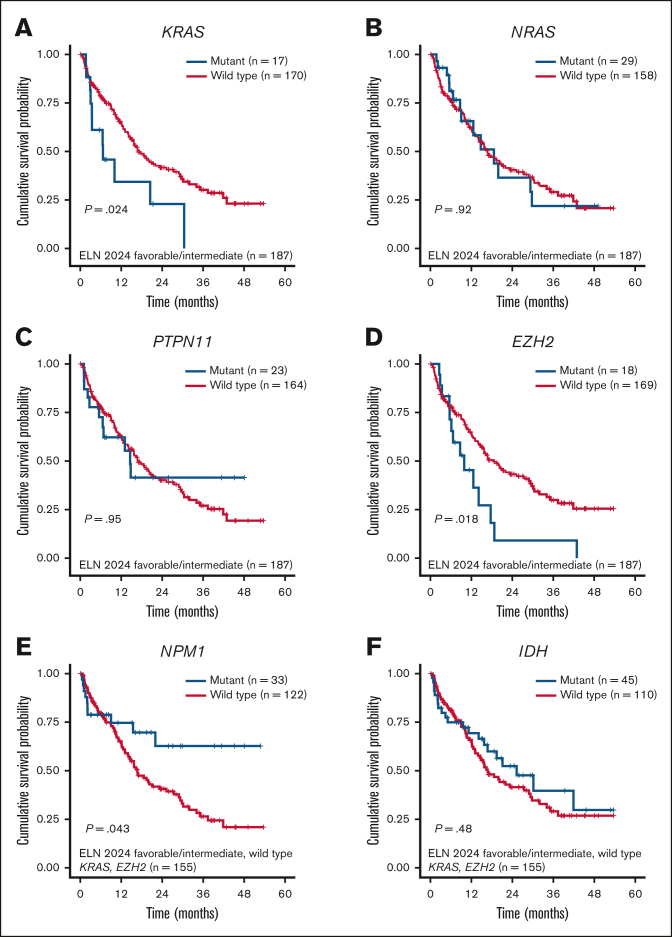
**Kaplan-Meier OS analysis for patients with newly diagnosed AML aged ≥60 years.** (A-D) Survival analysis stratified by *KRAS*, *NRAS*, *PTPN11*, and *EZH2* mutations among patients with ELN 2024 favorable- or intermediate-risk AML. (E-F) Survival analysis stratified by *NPM1* and *IDH* mutations among patients with ELN 2024 favorable- or intermediate-risk AML with *KRAS* and *EZH2* wild type.

