# Supplementary material for: Beat AML genetic risk stratification model in a cohort of older VEN/HMA-treated patients with AML
Source: Blood Neoplasia. 2025 Jun 3;2(3):100125. doi: 10.1016/j.bneo.2025.100125 (PMC12356277; doi:10.1016/j.bneo.2025.100125)
Supplement: Supplemental Tables and Figures [file BNEO_NEO-2025-000613-mmc1.pdf]

**Supplemental Tables and Figures**

**Supplemental Table S1**. Cytogenetics and most frequently mutated genes (n=238).

| | n (%) |
|---|---|
| **Cytogenetics**† | |
| Normal karyotype | 79 (33) |
| -5 or del(5q), -7 or del(7q), - 17/abn(17p) | 17 (7) |
| Inv(16) or t(8;21) | 3 (0.1) |
| Complex cytogenetics | 52 (22) |
| KMT2Ar | 6 (3) |
| Other | 51 (21) |
| Unknown | 30 (13) |
| **Mutations*** | |
| *SRSF2* | 66 (28) |
| *TET2* | 65 (27) |
| *RUNX1* | 61 (26) |
| *ASXL1* | 59 (25) |
| *TP53* | 51 (21) |
| *DNMT3A* | 50 (21) |
| *NPM1* | 41 (17) |
| *IDH2* | 40 (17) |
| *NRAS* | 34 (14) |
| *PTPN11* | 29 (12) |
| *U2AF1* | 22 (9) |
| *FLT3-ITD* | 22 (9) |
| *IDH1* | 21 (9) |
| *KRAS* | 20 (8) |
| *SF3B1* | 19 (8) |
| *EZH2* | 18 (8) |

[*]Mutations were considered present at any detectable variant allele frequency (VAF)

[†]Cytogenetics were available for 208/238

**Supplemental Table S2**. Risk classification assignment for each risk model

| | **Favorable-risk** | **Intermediate-risk** | **Adverse-risk** |
|---|---|---|---|
| **4-gene mPFS[1]** | 128 (53.8) | 59 (24.8%) | 51 (21.4%) |
| **Refined ELN 2024[2]** | 42 (17.6) | 109 (45.8%) | 87 (36.6%) |
| **Beat AML genetic risk model** | 61 (25.6%) | 94 (39.5%) | 83 (34.9%) |

1. Dohner H, Pratz KW, DiNardo CD, Wei AH, Jonas BA, Pullarkat VA, et al. Genetic risk stratification and outcomes among treatment-naive patients with AML treated with venetoclax and azacitidine. Blood. 2024;144(21):2211-22

2. Lachowiez CA, Ravikumar VI, Othman J, O'Nions J, Peters DT, McMahon C, et al. Refined ELN 2024 risk stratification improves survival prognostication following venetoclax-based therapy in AML. Blood. 2024;144(26):2788-92

**Supplemental Table S3**. Univariate analysis among mPFS favorable- and intermediate-risk groups (n=187) (i.e., all patients excluding *TP53* mutated)

| **Variable*** | **n** | **HR** | **95% CI for HR** | **p** |
|---|---|---|---|---|
| *ASXL1* | 54 | 1.459 | 0.97-2.19 | 0.067 |
| *BCOR* | 25 | 0.956 | 0.53-1.71 | 0.879 |
| *DNMT3A* | 48 | 0.613 | 0.38-0.98 | **0.042** |
| *EZH2* | 18 | 1.967 | 1.11-3.48 | **0.020** |
| *FLT3.ITD* | 21 | 0.937 | 0.49-1.80 | 0.844 |
| *IDH* | 54 | 0.824 | 0.53-1.28 | 0.391 |
| *KRAS* | 17 | 2.042 | 1.09-3.84 | **0.027** |
| *NPM1* | 40 | 0.654 | 0.38-1.13 | 0.128 |
| *NRAS* | 29 | 0.971 | 0.55-1.71 | 0.919 |
| *PTPN11* | 23 | 0.979 | 0.52-1.83 | 0.946 |
| *RUNX1* | 58 | 1.223 | 0.81-1.84 | 0.332 |
| *SF3B1* | 12 | 1.644 | 0.75-3.58 | 0.211 |
| *SRSF2* | 64 | 1.211 | 0.81-1.81 | 0.347 |
| *STAG2* | 25 | 0.703 | 0.39-1.28 | 0.252 |
| *TET2* | 56 | 1.696 | 1.13-2.55 | **0.011** |
| *U2AF1* | 19 | 1.403 | 0.81-2.43 | 0.225 |
| *WT1* | 10 | 0.402 | 0.128-1.27 | 0.120 |

*Mutations were considered present at any detectable variant allele frequency (VAF)

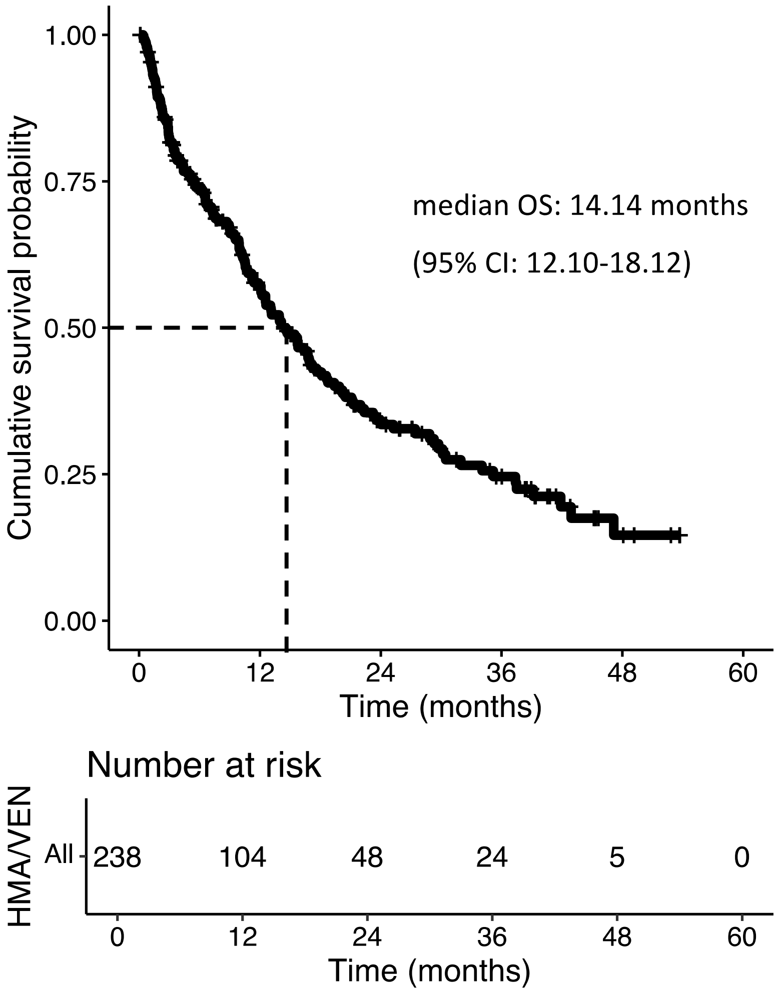


**Supplemental Figure S1**. Overall survival of all 238 patients with newly diagnosed acute myeloid leukemia 60 years or older treated with hypomethylating agents combined with venetoclax.

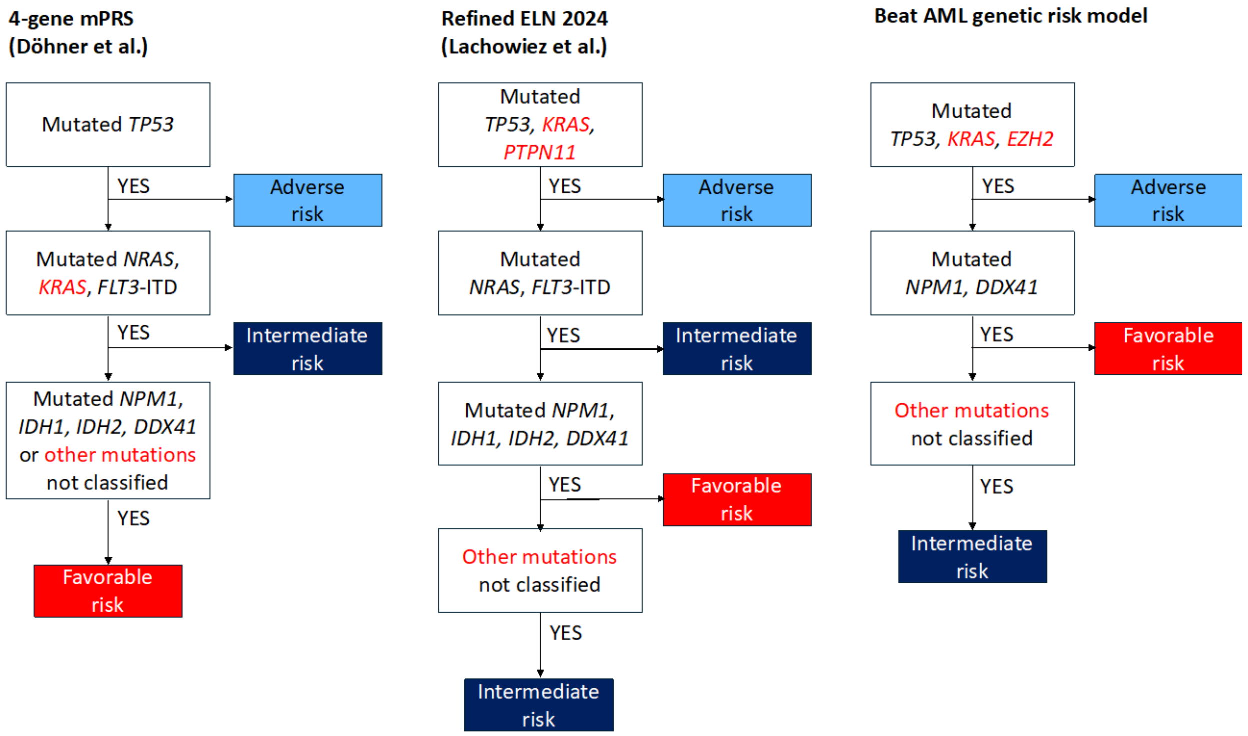


**Supplemental Figure S2**. Flow chart of the 4-gene mPRS by Döhner et al, the refined ELN 2024 by Lachowiez et al. and our refined Beat AML genomic risk model for patients treated with a hypomethylating agent with venetoclax.
